# Disparity in health care: HIV, stigma, and marginalization in Nepal

**DOI:** 10.1186/1758-2652-12-16

**Published:** 2009-08-26

**Authors:** Chandra K Jha, Jeanne Madison

**Affiliations:** 1 School of Behavioural, Cognitive and Social Science, University of New England, Armidale, New South Wales, Australia; 2 4/2 Prospect St, Glenroy, VIC 3046, Australia; 3 School of Health, University of New England, Armidale, New South Wales, Australia

## Abstract

**Background:**

The provision of effective health care to people with HIV and those from usually marginalised backgrounds, such as drug users and sex workers is a growing concern in Nepal, because these populations often do not seek health care, as willingly as the general population. Exploration of the factors, which hinder them seeking health care is crucial. The 'lived' experiences of the usually marginalized participants in this research will reflect on the constraining factors, and contribute to the development of appropriate strategies, which will facilitate people with HIV and other marginal populations to seek more readily appropriate health services.

**Methods:**

This study explored the healthcare-seeking experiences of 20 HIV-positive participants in Nepal, as well as 10 drug-using participants who had never had an HIV test and did not know their HIV status. Using grounded theory, this study investigated the perceptions and experiences of HIV-positive persons, or those perceived to be at risk for HIV, as they sought health care services in locations around Kathmandu Valley.

**Results:**

Health professionals were perceived to lack knowledge and sensitivity in providing health care to often marginalized and stigmatized injecting drug users, sex workers and HIV-positive people. Stigma and marginalization seem to interfere with doctors' and other health professionals' decisions to voluntarily treat persons who they perceive to be at high risk for HIV infections. Doctors and other health professionals appear suspicious, even unaware, of contemporary biomedical knowledge as it relates to HIV. The fear that certain marginalized groups, such as injecting drug users and sex workers, would be infected with HIV has further intensified stigma against these groups.

**Conclusion:**

The study identified the beginning of a change in the experiences of HIV-positive people, or those at risk of HIV, in their seeking of health care. With focused, contemporary HIV education and training, the beginning of positive changes in the knowledge base and attitude of health providers seemed to be apparent to some participants of this study.

## Background

The literature reveals that many health care providers still hesitate to provide care to people at high risk of HIV due to a fear of contracting HIV themselves [1-4]. Due to this fear, some doctors continue to refer people at high risk to other health facilities [1], transferring the perceived risk to other health professionals. Such fears are reported to be higher in health care providers who tend to be involved in invasive medical and surgical procedures [5-7]. Interestingly, however, doctors experienced and frequently involved in providing treatment and care to HIV patients perceive that there are few risks of HIV transmission [8,9] when appropriate precautions are in place.

Green and Platt [10] argue that people with HIV and their relatives and care givers have experienced stigmatization by health care providers. Such stigma affects day-to-day family and social relationships. For example, Richardson and Bolle [11] and Green and Platt [10] report the case of a doctor requiring that an HIV patient use a separate toilet in a hospital to control the transmission of infection. Other studies [10,12-14] report that people with HIV, admitted to health institutions for treatment, are isolated, even when it involves the use of eating utensils. Such experiences discourage people with HIV from seeking care from health providers.

A breach of confidentiality by health care providers has been illustrated in other studies [4,10,15,16]. For example, one drug user in Green and Platt's study [10] said that a general practitioner disclosed his HIV status to his mother without his permission or informing him later. Another patient said that his file was left exposed on a desk with an "HIV-positive" tag attached. In another instance, a patient in a general ward related being asked loudly by a nurse to take AZT in front of other patients, which was a breach of confidentiality.

Another patient [17] described how a doctor provided a patient's sero-positive status to the patient's employer without seeking consent from the patient. In yet another instance, a woman felt that a health worker was rude and uninformed: the health worker then referred her to a counsellor, who told the woman that she had AIDS rather than HIV [1]. The way counselling was provided was seen as ill informed and dissatisfying. For instance, the counsellor told her that she would not survive more than five years, and that her child would die in four months' time [1].

The number of people living with HIV has increased in Nepal. The first case of HIV was reported in 1988, and by 15 December 2008, the number of people with HIV had reached 12,933, of which 2151 had developed AIDS [18].

The use of HIV treatment guidelines have standardized medical practices, which seems to have contributed to decreased stigma against people with HIV in other countries [10,19,20]. In Nepal, the national strategy on HIV/AIDS included voluntary counselling and HIV testing in 2002 [21], and the national guidelines on antiretroviral therapy were introduced in 2004 [22,23]. These guidelines focus on the provision of quality medical care, antiretroviral drugs (ARVs), and voluntary counselling and HIV testing (VCT) services, including home-based and community-based care and supports [22-26]. The government of Nepal is initiating the training of health facility staff to improve the quality of care, including the use of universal precautions [24,25]. These plans are in the early implementation stages.

By and large, healthcare for people with HIV is limited to major hospitals in the Kathmandu Valley and a few regional hospitals [24,26]. Some non-governmental organizations (NGOs) also provide VCT services [27]. VCT services would be particularly helpful for the hidden populations, such as injecting drug users and sex workers, who are largely unable to access other intervention programmes [28]. Almost 100 NGOs are involved in education and prevention of HIV transmission. NGOs are major allies in implementing the government's HIV/AIDS programmes for targeted populations. These NGOs follow the national guidelines on HIV prevention.

However, the current national guidelines clearly reflect a western countries' context. Paradoxically, local cultural dynamics have major implications in the initiation of risk behaviours, such as drug use and sexual activities, leading to HIV transmission. Cultural factors also closely interlink with the process of stigmatization of people living with HIV and those practicing risk behaviours [29-31]. Almost all HIV/AIDS-related interventions in Nepal are funded by donor countries, bilateral agencies and the United Nations [32].

Nepal has a concentrated epidemic with the prevalence of HIV highest in at-risk groups, such as 68% in injecting drug users and 15.6% in street-based sex workers in Kathmandu Valley. In contrast, however, HIV prevalence is less than 1% in the general population. Although a generalized epidemic does not appear to currently exist, such contributing factors as seasonal migration, poverty, the exploitation of women and a lack of a nationally consistent response indicate high-risk factors for HIV transmission in Nepal's general population [24,26,33,34].

## Methods

This paper used grounded theory methodology to explore the experiences of people with HIV [35]. It reflects participants' points of views, and highlights how people with HIV perceive they are treated by health providers in Nepal's health care facilities. The study commenced in April 2004 and was completed in April 2006, concomitantly with the national reforms described in the literature and contextual review.

### Sample

Thirty participants were recruited for this study; of these, 20 were diagnosed with HIV, and 10 were injecting drug users who had never had an HIV test. The reason for including the 10 participants who did not know their HIV status was consistent with grounded theory, which encourages testing a phenomenon in diverse and contrasting ways [36-39]. For example, after understanding the experiences of drug users with HIV, the themes indicated the need to further investigate the experiences of drug users who did not know their HIV status. Was the experience and phenomenon described by people with HIV similar or different from other people at risk for HIV infection, such as drug users or sex workers? Thus, the interview process included at-risk informants to enrich the emerging themes [35,38,40].

Several categories emerged from the data, reflecting that health care providers initiated inadequate treatment and care to people with HIV. Data illustrated that besides an HIV diagnosis, high-risk behaviours, such as injecting drug use, also attracted stigma, leading doctors and other health care providers to withdraw, reduce or change the care they provided.

The ratio of male to female was equal. The participants were selected from a number of backgrounds, such as injecting drug users, women who had returned to Nepal from brothels in India, male migrant workers and housewives. The participants were selected by using purposive sampling. Support groups of people with HIV, NGOs, hospitals, and key persons who work in the field of HIV/AIDS and drug use were approached regarding the recruitment of participants. Kathmandu Valley, Hetauda, Narayanghaat and Pokhara were the selected study sites.

### Data collection

Data were collected by the use of in-depth interviews with the participants [41]. The interviews were one to two hours in length, and all were recorded on audiotapes. The sampling frame included: geographic coverage (urban versus rural); age group 18 to 50 years; male versus female; study populations (injecting drug users with HIV, injecting drug users without HIV, sex workers with HIV, and trafficked women with HIV); married versus unmarried; employed versus unemployed; living with family versus those not living with family; detoxified drug users versus those continuing drug use; HIV status revealed versus HIV status concealed; and symptomatic versus asymptomatic. Data collection was stopped once the identified themes reached theoretical saturation [38].

### Ethical consideration

Before commencing data collection, the researcher received ethics approval from the University of New England Ethics Committee. Considerations were given to maintain participants' privacy and confidentiality. Their voluntary participation was a priority. All the audiotapes or documents were coded with an alias, which was chosen by the participants.

### Data analysis

Consistent with grounded theory, the study explored the major themes from the collected data [35,38,42]. To develop themes, the researcher transcribed each interview from Nepali into English. A suitable "open code", which had corresponding meaning in the data, was collected and organized, together with similar codes by using axial coding techniques to show relationships among the various open codes. Selective codes were identified, which emerged as a central concept that brought various codes and categories under one heading [38,39,42]. Two themes, "role ambiguity: a doctor or a moral pundit" and "a trained doctor or an experienced quack", emerged, which highlighted the health care providers' responses toward people with HIV and other marginalized, high-risk groups, such as injecting drug users and sex workers.

## Results

### "Role ambiguity: a doctor or a moral pundit"

"Role ambiguity: a doctor or a moral pundit" was extracted as a leading theme from the participants' discourses. The role of doctors is "ambiguous" while interacting with or providing treatment to a patient with HIV or a member of other marginalized groups, such as drug users and sex workers, in hospitals. In regard to the care of people with HIV and those with perceived high-risk behaviours associated with HIV, doctors were highly judgmental towards patients' morality. The following findings support this theme, as reflected in excerpts from the narratives of the patients, Amar, Saroj, Dai, Punam and Sumitra.

Amar sought treatment for an abscess on his hand from a doctor in a Kathmandu hospital. During the consultation, he revealed his drug use, and the doctor recommended an HIV test. He was diagnosed with HIV and the doctors subsequently refused to treat him. Amar went to a nursing home, where he asked a doctor for treatment for his abscess. In the meantime, he presented all the reports of investigations he had had in hospital, including his HIV status. The doctors told him that if other patients knew about them providing care to a patient with HIV, other patients would suspect the doctor of also being HIV positive. The doctors were worried that the perception of them as "doctors with HIV" would undermine their social prestige and status, and would alienate them from society on the grounds of their HIV status or high risk for HIV.

After a refusal from hospital, I went to a nursing home with an abscess. A doctor said, "I do not see HIV patient." Other doctor said, "If we see the HIV patient, people will think we also have HIV." (Amar, p17)

From Amar's narratives, it is clear that the social stigma against HIV/AIDS is pervasive, even among educated and experienced professionals, such as doctors, who are expected to know about the transmission and prevention of HIV. At the same time, participants seem to be saying that they feel powerless against the existing social stigma around HIV/AIDS.

The doctors' medical knowledge, ethics and personal influence in society should play a crucial role in changing social stigma by disseminating facts about HIV, but many fail to fulfill this important public health role. On the one hand, refusing to provide care to a patient in itself humiliates people with HIV; on the other hand, the decision to not provide care perpetuates existing social stigma and misinformation.

Participants' narratives reveal that health care providers are not always professionally or ethically prepared to handle the issues associated with people with HIV or at risk of contracting HIV. A big dilemma for participants with HIV is whether or not to reveal their HIV status to doctors. Fearing that doctors may mistreat them, some hide their status. Doctors and other health care providers appear to be susceptible to the prevailing prejudice and fear toward HIV or people with HIV. Such attitudes and behaviours make the patients feel helpless, frustrated and humiliated. As a consequence, patients feel discouraged from seeking care or staying in the hospital.

According to participants, doctors have not overcome their bias against specific groups or marginal members of society, such as injecting drug users. They are perceived of as unacceptable or of little importance, which leads doctors to behave toward those patients in an aggressive or harsh manner.

As a strategy to avoid a doctors' possible negative reaction, Saroj, unlike Amar, initially decided not to disclose his drug use to doctors. Not disclosing relevant information complicated Saroj's treatment. When he did disclose this information, he was anxious, noting the doctors' changed behaviours and attitudes. Saroj related:

Once I had persistent fevers and convulsions in the evening. My mother took me to a hospital assuming that I got TB. Doctors were behaving well. Later I felt I wouldn't survive because my illness was badly progressed. So I felt [it] necessary to disclose my drug use. Then, they asked me to undergo HIV testing and other tests. After HIV diagnosis, they were talking aggressively with me. (Saroj, p4)

Saroj's narratives reflect conflict between the social versus medical roles of doctors in Nepal. For example, doctors are responsible for providing effective and compassionate care to a patient without prejudice. Recommending laboratory investigations is clinically appropriate and justifiable to help to diagnose and provide effective treatment. However, withdrawing services and becoming unfriendly following Saroj's disclosure of his drug use and his HIV diagnosis reveals stereotyping and prejudice. This led the doctors to behave more like "moral pundits" than "health experts or doctors".

According to the participants in this study, doctors and nurses appear to not respect the moral and ethical rights of marginalized patients, particularly those who are diagnosed with or at high risk of contracting HIV.

Dai mentioned that the behaviour of the health care provider seemed to breach the confidentiality of patients with HIV. He offered insight into how such behaviour served to humiliate people with HIV in a health care setting:

Doctors just disclose the [status of the] HIV infected persons. A woman was HIV positive. She was admitted to a hospital for delivery ... They put the name "positive" [on] both mother and baby. Nurses used to call them "positive". They shouldn't do like that. It damages people with HIV mentally ... (Dai, p19)

The narratives present an "institutionalized" humiliation in which the "powerful" ones impose their superiority over the "powerless" ones. A hospital is a particular institution in which doctors and nurses act as the powerful ones. Their role is crucial for the day-to-day running of the hospital, as well as the well being of patients. Although their job is highly respected and appreciated, it is not the case that their behaviour is always (or necessarily) beneficial to patients or that they display an acceptable manner when dealing with patients.

The label, "positive", used in a hospital for a patient with HIV, appears to be revealing and value laden. According to study participants, it would appear that doctors and nurses intentionally or unintentionally humiliate people with HIV. On the other hand, such humiliation may be associated with the patient's "powerlessness" in the hospital. The disadvantaged and marginalized lack the resources to protest against such humiliations.

Some participants stated that they were denied the provision of health care in a hospital by health care professionals. A reason is that most hospitals in Nepal are not well prepared to provide care to people with HIV. This highlights the need to address the training of doctors and nurses, as well as to ensure the institution is provided with a treatment protocol. Without training, doctors still seem to erroneously fear that HIV will be transmitted to them if they provide care to people with HIV. Punam, seeking treatment in a rural area, said:

We had face-to-face conversations, and the doctors used to say, "Is this hospital made for HIV infected people? Better you go to Kathmandu." [Punam, p3]

Punam's narratives highlight that peripheral health care facilities in Nepal are not equipped to treat people with HIV. In fact, many patients simply cannot travel to Kathmandu from the rural areas for treatment; there is a clear gap in services in the rural health care system.

According to Sumitra, some doctors appear to be judgmental toward patients with HIV. Participants talk about doctors who seem to feel that providing treatment to HIV-positive people is a waste of resources. When doctors do provide care, participants describe it as failing to be compassionate and enthusiastic. Such attitudes seem inappropriate for health professionals, and have deleterious implications for the survival of people with HIV.

In hospital, doctors perceive that people with HIV will die ultimately; they will not survive; and they feel it is useless to provide care to people with HIV. [Sumitra, p3]

Sumitra's narratives reveal that the "poor prognosis" for HIV/AIDS seems to reduce aggressive and appropriate care by health professionals. People with AIDS need critical, intensive and expensive care for longer periods compared to people with other diseases; AIDS-related illnesses are prolonged and recurrent. This appears to create frustration in health providers and fosters a belief that there is no benefit in providing care to people with AIDS.

### "A trained doctor or an experienced quack"

Some participants revealed that doctor's attitudes, skills and ethical code of practices were the major obstacles in the health care setting to the provision of HIV/AIDS-related care. Some doctors demonstrate poor knowledge, failing to strictly follow universal precautions, such as the use of aseptic techniques and wearing gloves. Failing to wear gloves means that there is a high chance of transmitting various diseases; in other words, doctors place themselves and other patients at risk of acquiring infections from patients.

Despite having this critical knowledge, which doctors learn during their basic medical education, such behaviour has led to people wondering whether they are "trained doctors or experienced quacks". This issue has emerged as a dominant theme. As Sharad, a lay observer, stated:

Doctor was trying to squeeze his wound with bare hands ... I [said], "Please don't do that with open hands. Wear gloves." He asked me, "Why do you pretend knowing more?" I [said], "It's not a matter of knowing more ... he is HIV positive. If you have a wound in your hand, it might transmit [to] you too." The doctor said, "Why didn't you tell me before that he got AIDS? Do this dressing yourself." He threw medicines at me. (Sharad, p27)

Such an egotistical refusal to comply with professional standards is counterproductive to the appropriate treatment of HIV-positive patients and all other patients. Mal-appropriate practices, such as this, not only undermine patients and their treatment, but the doctors also place themselves at a greater risk of acquiring and transmitting infections. The role of doctors as health care "experts" here is arguable. When Sharad suggested that the doctor use universal precautions, it seems that he perceived this as a slur on his reputation. This led the doctor to insult the patient and the patient's attendants, and remarkably, to then refuse to apply a dressing.

In contrast to Sharad's observations, some participants revealed that certain hospitals stressed the provision of care to people with HIV without discrimination. Although doctors have some degree of fear about HIV transmission, such a policy has coerced them into providing care to people with HIV. However, the doctors seem overly cautious.

In hospital, it didn't happen to me, but to one of my friends. Doctors didn't want to touch him. Doctors wore three gloves to give a saline water drip. (Rajesh, p1)

In these narratives, fear and obligation emerge as part of doctors' care of patients with HIV. Although some doctors seem uninterested or uneasy in providing care to people with HIV, in some cases they feel morally or ethically obliged to care for them. According to Rajesh, a doctor was afraid of contracting HIV from the patient and wore three pairs of gloves while providing care. Such a practice creates unnecessary fear in patients with HIV and other observers, creating a sense that people with HIV pose a threat to others.

In a case related by Sumitra, a patient's support group became dissatisfied with the doctors' manner of treatment, deciding to take the patient home to be cared for by family members. This suggests that, in the light of potential humiliation faced by people with HIV in the Nepal health system, home care is an alternative.

The patient's relatives and support group had to face significant difficulties. Later, the patient family and support group argued with them [hospital staff], and said that [the] patient will get better care and love at home than ... in hospital. Although the patient should have stayed for a month, they took the patient home in two to three days. (Sumitra, p5)

### The beginning of compassionate health care

Although health care has been criticized as often discriminatory against people with HIV, as we have described, some recent experiences suggest that the provision of health care is beginning to improve. For example, Sumitra noted a change in the doctors' manner and attitudes toward her. Although her past experiences were not good, she now feels that the doctors seem ready to admit her to hospital and provide care. The doctors interacted with her and gave psychological support, such as compassion, hope and encouragement about her life expectancy. This made her more optimistic, giving her the impression that doctors cared about her:

This time, they kept beds tidy. Doctors [were encouraging, saying] that nothing will happen; you are very well now; you will survive longer ... We got lots of encouragements from doctors. In Patan Hospital, both native and foreign doctors used to care for people with HIV, and touch them gently. (Sumitra, p5)

Such a change in attitudes and provision of care by doctors toward people with HIV is a sign of possible improvement in the Nepalese health care system. However, this change appears limited to very few hospitals and very few doctors.

For example, Patan Hospital is a mission hospital, and has been managed by doctors and specialists from western countries; it appears that these doctors are aware of the policies and protocols related to HIV treatment. Beine [29] cites the letter of Mark Zimmerman, Director of Patan Hospital, to all hospital staff, in which he says that care provided to patients with HIV must be equal to that of all other patients. He notes that this includes maintaining confidentiality and strict universal precautions for all patients. This appears to be a step forward in the implementation of HIV treatment policy. However, such directives could not be found in other hospitals.

A patient, Rajesh, identified significant distress due to his HIV diagnosis and described his experience of visiting a specialist in HIV medicine. Rajesh was impressed by his treatment and counselling: he felt relief from his anxieties as a direct outcome of the way the doctor counselled him.

He counselled me so nicely. Listening to his suggestions and counselling I felt nothing has happened to me. (Rajesh, p3)

Some participants described an improvement in the level of understanding of health providers who seem to be developing their skills to provide both clinical care and emotional support to people with HIV. This has helped participants, such as Sumitra and Rajesh, to develop a more hopeful and positive attitude toward doctors.

In Sarita's experience, the doctor provided good counselling before providing her with her HIV-positive result. She also felt that the counselling was very useful in assisting her to cope with HIV.

Doctor told me, "You are HIV positive". Doctor didn't hate me. Doctor was telling me good things ... nobody will die soon. A disease can happen to anyone, either rich or poor. People can live with HIV longer. (Sarita, p8)

Sarita's doctor focused on the major issues associated with an HIV diagnosis, such as counselling, providing detailed information about coping with HIV, approaches to maintaining health, and de-stigmatizing HIV. This suggests that hospitals and health providers may be preparing to provide holistic care to people with HIV. The extent and quality of this possibly improved care, however, is yet to be assessed.

For Sapan, the doctors even seemed to recognize and empathize with his circumstances when he sought care for tuberculosis. In Nepal, the direct observed short course treatment is the regimen of choice for tuberculosis, and it requires that the patient visit the doctor every day to receive treatment. Sapan revealed his HIV status to his doctor, indicating that he would not be able to undertake travel for daily treatments; the doctor organized treatments that Sapan could use at home, which meant that he would have to go to the hospital weekly, rather than daily.

While seeking treatment for tuberculosis, I was thinking [of] whether to tell or not about my HIV diagnosis, but I told later when the doctor asked me whether I had any other disease. Nothing happened so far. Others had to come daily for the medicine, but I had to come once a week. It was easy for me. (Sapan, p21)

Sapan's case indicates that stigma might be decreasing in health care settings. Doctors seem to be considering various approaches and strategies regarding how care can conveniently be provided to a patient with HIV.

Traditionally, Nepal's doctors limit their practice to examining patients and prescribing certain medicines. People with HIV face a number of psychosocial and cultural issues that require much more "holistic care". Medicine alone is not sufficient to heal them. These issues can be mitigated by enhancing doctors' skills in interacting with HIV patients, providing counselling, and demonstrating positive attitudes towards their patients. This holistic approach should improve the quality of health care services, effectively helping to improve the health and longevity of patients with HIV.

## Discussion

The image of the HIV virus automatically leading to death was prevalent in the early stage of the global pandemic [43-46]. The implications of such a perception on the day-to-day lives of people with HIV are grave; this includes how they utilize health services. This study focused on reviewing the provision of health care from a cultural and biomedical perspective in the hope of providing more insights into how cultural and social stigma conflicts with the provision of quality health care to people perceived to be at high risk for HIV.

Stigma among doctors and other health care providers appears to be grounded in culture, which ultimately compromises the treatment and care of people with HIV and members of other related marginal groups, particularly drug users and sex workers.

The genesis of stigma among doctors and other health workers is a cultural experience [47-49]. People develop ingrained negative thoughts about drug use and prostitution from childhood, and for health professionals, this occurs long before they acquire their health education and qualification. The prevailing cultural model includes the notion that the best way to avoid HIV transmission is simply self control or eliminating behaviours that put people at risk of HIV transmission.

Accordingly, diagnosis or being at risk of contracting HIV is perceived as punishment for "violating" cultural norms. In such instances, doctors feel that providing treatment to people with HIV or at-risk people also violates cultural norms. As illustrated in this study – particularly in the doctors' remarks that people would suspect them of being HIV positive if they treated Amar – some doctors protect their identity and high social standing by declining to treat people with HIV. As reflected in Dai's narratives, health providers labelling a mother and her newborn baby as "HIV positive" would be perceived as an abuse of (health provider) power.

This study reviewed the health care experiences of people with HIV or at risk of contracting HIV from a biomedical perspective, and found the medical response disappointing. The phobia associated with the virus and the fear of its transmission has complicated health providers' decisions to willingly treat people with HIV. Despite the fact that universal precautions are effective in curbing HIV transmission, as per widely accepted infection control guidelines, many doctors and nurses obviously remain unconvinced of their efficacy. This is evident in Rajesh's narrative that a doctor was overly cautious and wore three pairs of gloves while giving intravenous saline.

This finding is congruent with the study of Kermode *et al *[2], which reveals that a large majority of health care workers in rural India perceive that they have high chance of acquiring HIV simply through providing care to patients with HIV. In the absence of universal precautions, needle stick injuries and invasive medical or surgical procedures [2] are correctly seen as potentially high-risk routes for transmission of HIV. However, other studies [50-52] report that such fears are decreasing with the use of treatment protocols and guidelines, training of health care providers, and equipping health facilities with appropriate resources and equipment. Nepal often lacks such protocols, education and resources. Health care providers' refusal to care for Punam demonstrates that Nepal does not yet have appropriate strategies in place.

From the mid-1990s, international responses and supports were effective in Nepal, and resulted in the formulation of policies to manage HIV/AIDS [24,33,34]. However, according to the participants in this study, implementation, monitoring and evaluation of those policies and strategies still seem to be exceptionally weak. For instance, just one hospital, Patan, in the centre of Kathmandu Valley, had a written directive from the hospital director that patients with or at risk of HIV should receive the same care as all other patients. Health care providers were also directed to follow strict universal precautions to control transmission of infections.

This study's participants perceived that health providers seemed to believe that health care was not appropriate for people with HIV because they were going to die. In such cases, the participants felt that health providers were reluctant to pursue expensive treatment to people with HIV as it is seen as an unnecessary investment. This is a critical finding, which is linked to the importance of sensitive counselling and palliative care needs.

When an HIV patient does not respond well to treatment and has progressed to a terminal stage, much can be done through the provision of comfort measures and pain relief to maintain his or her dignity [53,54]. Delivering this level of health care will require significant education and resources.

This study indicates that health as a fundamental human right [55] has not yet been realized in Nepal. This has altered the relationship of health care providers and health seekers in the process of health care consultation. The addition of stigma and marginalization attached to people with or at risk of HIV further harms the dignity of people seeking health care. At-risk people seeking health care are in a weak position to proactively discuss their health issues compared to patients who are not at risk. For these participants, the stigma and marginalization associated with HIV seems to reduce the mutuality of health care decisions. Doctors seem to dictate to people from marginalized groups, instead of discussing plans for health care.

Apart from this weakness, this study noted signs that doctors' response towards people with HIV has started to improve. In recent years, focusing on health providers' training in care provision to people with HIV seems to have had some crucial and positive effect on doctors' attitudes, knowledge and skills. Other studies [10,56] have illustrated such changes in doctors' mindsets and practices, and subsequently, improvements in the quality of care to people with HIV after the provision of specialized training in HIV/AIDS. This study identifies that educating doctors in counselling skills, compassion and the provision of encouragement to HIV patients has improved the coping abilities of people with or at risk of HIV.

## Conclusion

The study demonstrates how cultural and biomedical aspects compete in the health care setting. The dominance of long-held cultural values by health care providers seems to have resulted in stigmatization of people with or at risk of HIV, as well as of people in other marginal groups, such as drug users and sex workers. As a consequence, at-risk people suffer from poor health and health care. Lack of knowledge and entrenched cultural attitudes has increased fear on the part of health care providers in Nepal. Despite the wide acceptance of the effective use of universal precautions in stopping the transmission of HIV and other blood-borne diseases, health care providers continue to avoid or refuse to care for at-risk populations.

Health care providers seem to persist in the belief that HIV automatically assumes a poor prognosis, discouraging doctors from treating people with or at risk of HIV. Efforts should be made to organize health care responses to marginalized, at-risk groups.

A recommendation from this study is the proactive enforcement and monitoring of the fulfillment of the "right to health" for people with HIV, as for all other patients. A task force committee consisting of health care providers, public health experts, hospital management and other key persons, including representatives of people with HIV and other marginal groups, should be formed to appraise and ensure that health services are appropriate and accessible for people with and at risk for HIV. Such an environment will encourage people with HIV to seek and receive health care without fear. Likewise, curricula related to marginalization and stigma should be developed and included in the training of all health care providers.

## Competing interests

The authors declare that they have no competing interests.

## Authors' contributions

CKJ, with professional expertise in HIV/AIDS study and research designed the study, conducted interviews and analysis of the data, and prepared the manuscript. JM provided inputs on this research and edited the manuscript. All authors have read and approved the final manuscript.
